# Hemifacial Spasm Caused by a Remote Tentorial Meningioma: A Case Report and Review of the Literature

**DOI:** 10.7759/cureus.106255

**Published:** 2026-04-01

**Authors:** Sreeraj K, Sridhar K, Emmanuel Thas, Satish Kannan, Christie Bernard

**Affiliations:** 1 Neurosurgery, Kauvery Hospital, Chennai, IND

**Keywords:** hemifacial spasm, meningioma, posterior cranial fossa tumour, remote tumours, spontaneous resolution

## Abstract

A 24-year-old female presented with right-sided hemifacial spasm (HFS). Imaging showed a right tentorial meningioma. The tumour was causing mass effect over the cerebellum. She underwent a right suboccipital craniotomy and excision of the lesion. Intraoperatively, it was found that the flocculonodular lobe was plugging the internal auditory meatus, thereby compressing the VII-VIII complex. Following surgery, she had complete relief of HFS. Remote tumours causing HFS are a rare phenomenon, with only a few cases reported in the literature. The causes of this phenomenon have been debated. In our patient, the tumour-related mechanical effect is the most probable mechanism for the HFS. The authors feel that an additional microvascular decompression may not be required in these cases. Remote tumours causing HFS are a rare phenomenon. To our knowledge, we report the fifth case in the literature of a remote tentorial meningioma causing HFS, with a review of the literature.

## Introduction

Hemifacial spasm (HFS) is a condition in which there are recurrent involuntary contractions of facial muscles on one side. The underlying mechanism is thought to be an ectopic excitation and ephaptic transmission between facial nerve fibres, most often due to vascular compression at the root exit zone (REZ) of the facial nerve [1]. In about 0.3%-0.6% of cases, a lesion in the cerebellopontine angle (CPA) can cause HFS [2,3]. It is extremely rare for a tumour located remote from the CPA to cause HFS. The English literature has only 14 previously reported cases [4-17].

In this report, we present a case of a giant tentorial cerebellar convexity meningioma causing ipsilateral HFS; to our knowledge, this is the fifth case of a tentorial meningioma causing HFS in the literature.

## Case presentation

A 24-year-old female presented to the Outpatient Department with a history of intermittent twitching of the right side of the face for the past five months. Initially, she had twitching of the right eyelid three to four times a day, which gradually increased in frequency and later started involving the right perioral region as well. The intensity and frequency of the HFS progressively increased and, on presentation, involved the whole of the right side of the face. She did not have any other symptoms, such as imbalance or decreased hearing.

Neurological examination showed right-sided HFS, which was spontaneous and increased on forced eye closure. Fundus and other cranial nerves were normal, including hearing and the lower cranial nerves. There were minimal right cerebellar signs, such as impaired finger-nose-finger test and dysdiadochokinesia.

**Figure 1:**
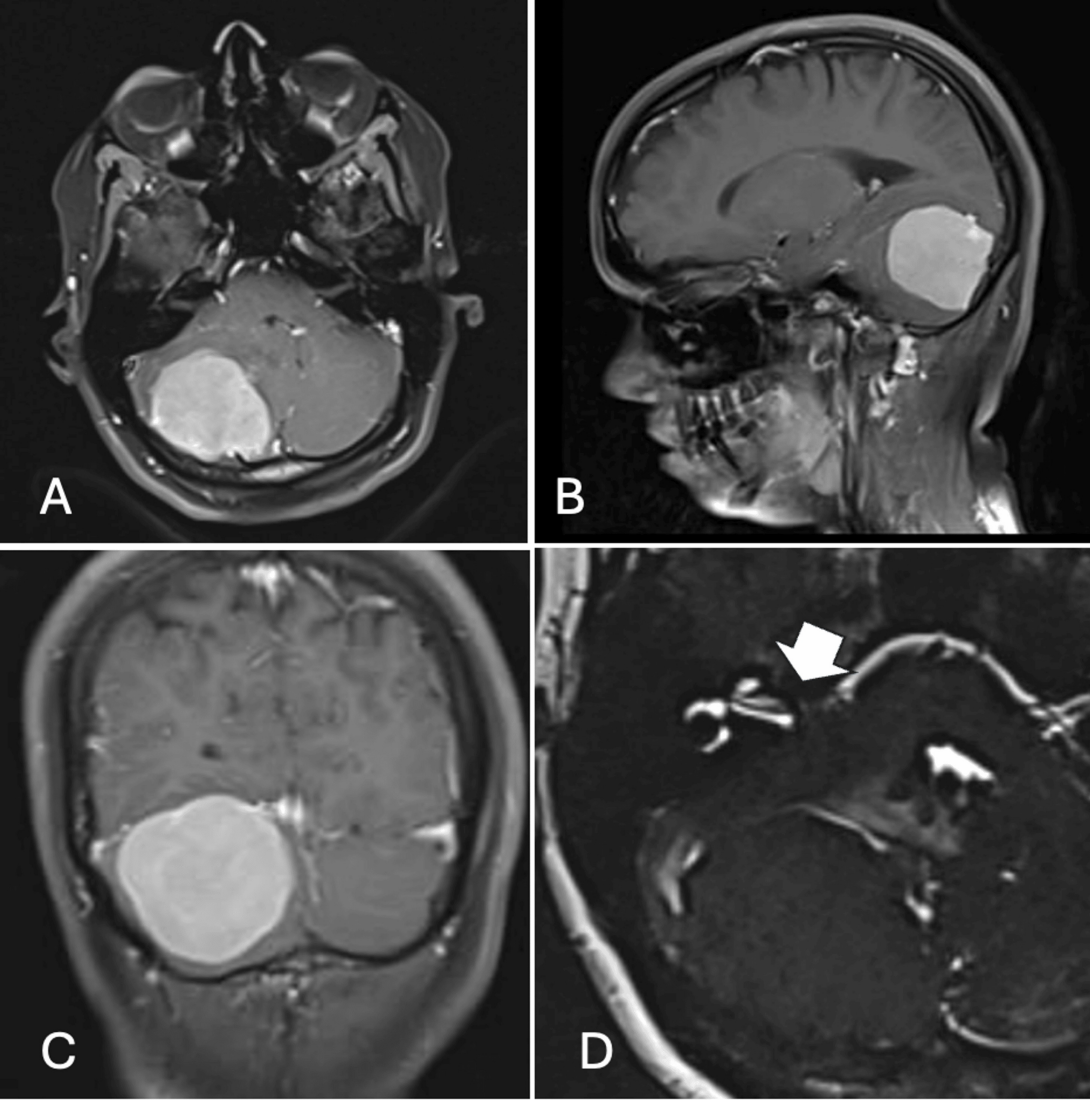
MRI of the brain A) Axial T1-weighted post-contrast image, showing a giant homogeneously enhancing mass in the right cerebellar hemisphere. B) Sagittal post-contrast image, showing convexity attachment of the mass and ventroinferior displacement of the cerebellum. C) Coronal post-contrast image, showing the tentorial attachment of the mass. D) Axial CISS image, showing that the right VII-VIII complex (white block arrow) is kinked and stretched by the flocculonodular lobe of the cerebellum, while the left side is free. CISS: Constructive Interference in Steady State

She was evaluated with an MRI of the brain, which showed an extra-axial lesion measuring about 4.7 × 4.4 × 4.3 cm, dural-based and attached to the inferior aspect of the tentorium and cerebellar convexity on the right side (Figure 1). The tumour was hypointense on T1-weighted and T2-weighted images, with intense homogeneous contrast enhancement, causing mass effect on the fourth ventricle. The VII-VIII complex on the right side was displaced anteriorly due to the mass effect of the lesion. The imaging features were suggestive of a meningioma. Pure tone audiometry was normal.

**Figure 2:**
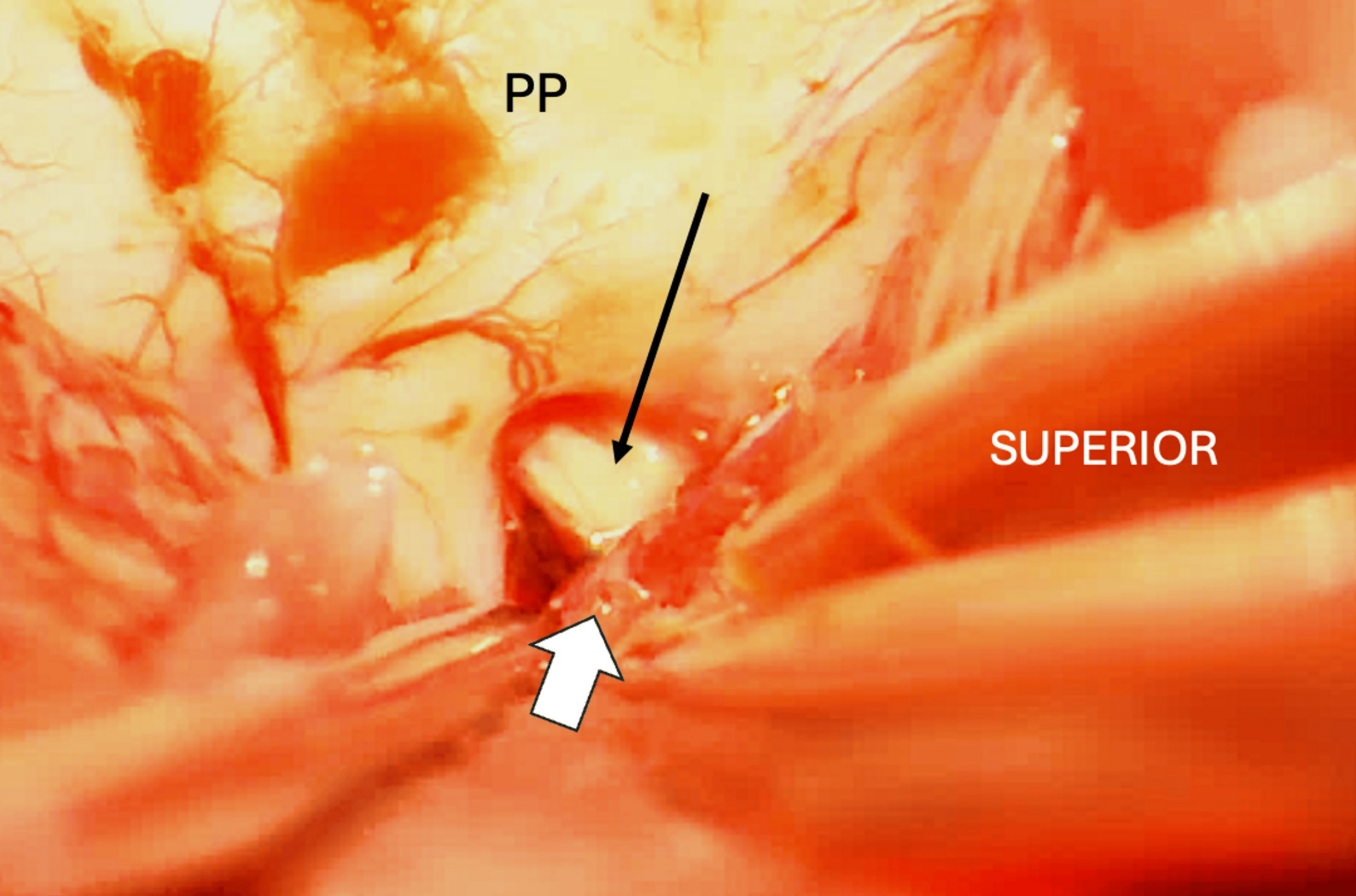
Intraoperative microsurgical view of the right cerebellopontine angle Intraoperative microsurgical view of the right cerebellopontine angle, showing the VII-VIII complex (thin black arrow) entering the internal acoustic meatus, and compressed by the flocculonodular lobe of the cerebellum (white block arrow). PP: Posterior Petrous Bone

**Figure 3:**
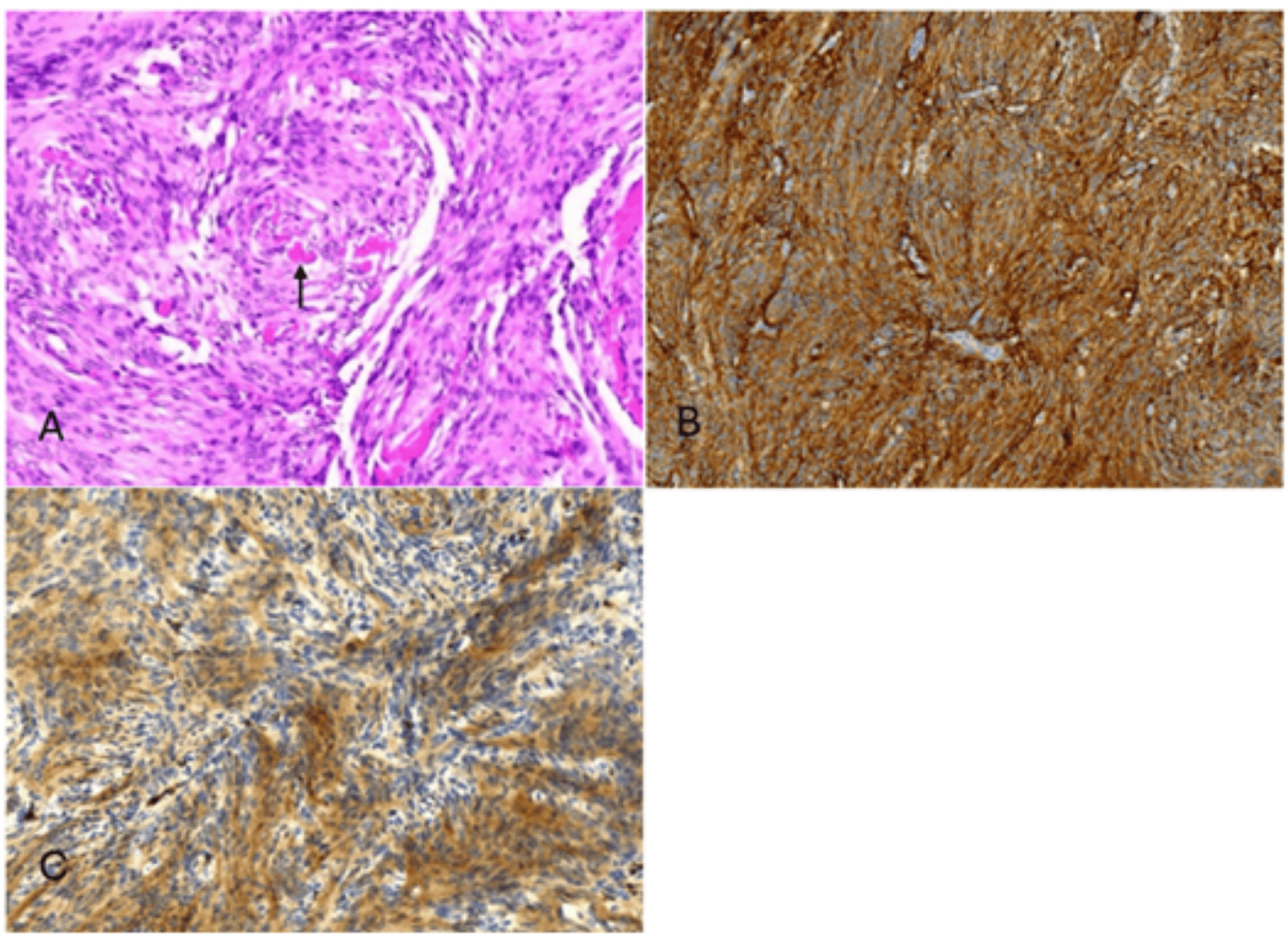
Histopathology and immunohistochemistry of the lesion A) The histopathological examination showed syncytial sheets of meningothelial cells with psammoma bodies (arrow). Immunohistochemistry showed expression of EMA and SSTR2A (B and C).

The patient underwent a right suboccipital craniotomy and complete excision of the lesion using microsurgical techniques. Intraoperatively, on inspection of the CPA, it was found that the flocculonodular lobe of the cerebellum was plugging the internal auditory meatus, causing kinking of the VII-VIII nerve complex, and there was no vascular conflict noted (Figure 2). Histopathology confirmed the diagnosis of meningioma (Figure 3).

The patient reported a significant reduction in the intensity and frequency of the spasm on the first postoperative day, and was completely symptom-free on follow-up, 10 days post-surgery.

## Discussion

Hemifacial spasm is a condition in which there are recurrent involuntary contractions of the muscles of facial expression on one side. The underlying mechanism is thought to be due to a neurovascular conflict between the facial nerve and an artery - most often the anterior inferior cerebellar artery [18,19]. Secondary HFS can arise due to other pathologies in the posterior fossa involving the CPA, e.g., meningioma, epidermoid, arteriovenous malformation, and aneurysms close to the facial nerve [2,3,20,21].

It is extremely rare for HFS to occur in a patient with a tumour away from the CPA, that is, with no direct contact with the facial nerve. The English literature has 14 such cases reported (Table 1). They are all individual case reports and include fourth ventricular tumours (four), contralateral CPA tumours (two), foramen magnum lesions (two), and a cerebellar dermoid (one). Bhayani and Goel reported a supratentorial meningioma causing HFS [7]. Interestingly, there were only four tentorial meningiomas causing HFS in the literature [14-17]. Several mechanisms have been proposed to explain this phenomenon of remote tumours causing HFS, including deformation or stretching of the facial nerve root, worsening of a previously silent neurovascular conflict, and alterations in the venous drainage or congestion due to tumour-induced displacement of posterior fossa structures [15]. HFS due to involvement of the facial nerve nucleus in the floor of the fourth ventricle has been postulated to be the cause in cases of fourth ventricular tumours causing HFS [5].

**Table 1:** Remote tumours causing HFS HFS: Hemifacial Spasm

S. No.	Author/year	No. of cases	Tumour	Side	Age	HFS recovery
1	Langston and Tharp (1976) [4]	1	Ganglioglioma of the fourth ventricle	Left	6 weeks/M	Transient recovery
2	Bills and Hanieh (1991) [5]	1	Ganglioglioma of the fourth ventricle	Left	2/M	Immediate postop
3	Rhee et al. (1995) [6]	1	Contralateral CP angle meningioma	Right	40/F	Immediate postop
4	Bhayani and Goel (1996) [7]	1	Falcine meningioma	Right	50/M	Postop
5	Matsuura and Kondo (1996) [8]	1	Contralateral vestibular schwannoma	Left	55/M	3 weeks
6	Sandberg and Souweidane (1999) [9]	1	Fourth ventricle pilocytic astrocytoma	Right	11/M	Reduced intensity and frequency
7	Harrison et al. (2000) [10]	1	Ipsilateral lower clival and foramen magnum meningioma	Right	63/F	Several days postop
8	Rossetto et al. (2011) [11]	1	Fourth ventricle ependymoma	Left	45/F	Immediate postop
9	Matsuda et al. (2016) [12]	1	Foramen magnum meningioma	Right	80/M	Postop
10	Hirayama et al. (2023) [13]	1	Cerebellar hemisphere dermoid cyst	Left	48/M	Immediate postop
11	Ogasawara et al. (1995) [14]	1	Tentorial meningioma	Right	79/F	Recovered in 6 months
12	Cancelli et al. (2005) [15]	1	Tentorial meningioma	Left	45/F	Recovered in 3 months
13	Park et al. (2009) [16]	1	Tentorial meningioma	Right	34/F	Recovered in 3 weeks
14	Nayak et al. (2022) [17]	1	Tentorial meningioma	Left	51/F	Recovered in 3 days
15	Current Study	1	Tentorial meningioma	Right	24/F	Recovered in 10 days

Tentorial meningiomas causing HFS are very rare, with only four previous cases reported in the literature. The cases did not have any age, sex, or side predilection. The tentorial meningioma in our case was large and caused mass effect on the cerebellar hemisphere. This resulted in secondary compression and distortion of the ipsilateral VII-VIII complex by the flocculonodular lobe. It is likely that excision of the lesion relieved the mass effect in the posterior fossa, thereby relieving the compression-distortion of the nerve. This was evident from the fact that, following complete tumour excision, the intensity and frequency of HFS were reduced on the first postoperative day, completely disappeared on the 10th postoperative day follow-up, and remained symptom-free until the last review, three months post-surgery. Hirayama et al. [13], in their case, not only excised the tumour but also performed a microvascular decompression of the facial nerve, as there was a vessel causing neurovascular conflict. We did not think that this step was needed for our patient.

## Conclusions

HFS can be a symptom of a tumour that is in direct contact with the facial nerve in the CPA, but it could also occur as a symptom of a distant tumour. To our knowledge, our case is the 15th case in the literature demonstrating HFS due to a distant brain tumour, and the fifth due to a distant tentorial meningioma. Resolution of HFS once the tumour is removed supports the relationship between the tumour-related mechanical effect on the facial nerve and the patient’s HFS. These patients may not require an additional microvascular decompression procedure.
